# Additive-Manufactured Gyroid Scaffolds of Magnesium Oxide, Phosphate Glass Fiber and Polylactic Acid Composite for Bone Tissue Engineering

**DOI:** 10.3390/polym13020270

**Published:** 2021-01-15

**Authors:** Lizhe He, Xiaoling Liu, Chris Rudd

**Affiliations:** 1 International Academy of Marine Economy and Technology, University of Nottingham Ningbo China, Ningbo 315100, China; Lizhe.He@nottingham.edu.cn; 2 Faculty of Science and Engineering, University of Nottingham Ningbo China, Ningbo 315100, China; 3 New Materials Institute, University of Nottingham Ningbo China, Ningbo 315100, China; 4 College of Science and Engineering, James Cook University, 149 Sims Drive, Singapore 387380, Singapore; Chris.Rudd@jcu.edu.au

**Keywords:** additive manufacturing, phosphate glass fiber, polylactic acid, gyroid, bone tissue engineering scaffold

## Abstract

Composites of biodegradable phosphate glass fiber and polylactic acid (PGF/PLA) show potential for bone tissue engineering scaffolds, due to their ability to release Ca, P, and Mg during degradation, thus promoting the bone repair. Nevertheless, glass degradation tends to acidify the surrounding aqueous environment, which may adversely affect the viability and bone-forming activities of osteoblasts. In this work, MgO was investigated as a neutralizing agent. Porous network-phase gyroid scaffolds were additive-manufactured using four different materials: PLA, MgO/PLA, PGF/PLA, and (MgO + PGF)/PLA. The addition of PGF enhanced compressive properties of scaffolds, and the resultant scaffolds were comparably strong and stiff with human trabecular bone. While the degradation of PGF/PLA composite induced considerable acidity in degradation media and intensified the degradation of PGF in return, the degradation media of (MgO + PGF)/PLA maintained a neutral pH close to a physiological environment. The experiment results indicated the possible mechanism of MgO as the neutralizing agent: the local acidity was buffered as the MgO reacted with the acidic degradation products thereby inhibiting the degradation of PGF from being intensified in an acidic environment. The (MgO + PGF)/PLA composite scaffold appears to be a candidate for bone tissue engineering.

## 1. Introduction

Bone tissue engineering (BTE) scaffold is the biomaterial construct that serves as the substrate to induce and facilitate the regeneration of bone tissue [1]. In terms of biomaterial engineering, the BTE scaffold shall be designed and prepared into a well-defined porous construct using the properly processed biomaterials, as both the structural and material properties dictate the actual performance of a BTE of restoring the original structure and function of a defected bone [2,3,4].

Gyroid surface received increasing interest in recent years as a scaffold architecture, owing to a continuous surface that supports colonization of osteogenic cells [5,6], controllable pore size that may balance the angiogenesis of regrown bone and load-bearing performance [7,8], as well as high porosity that facilitates mass transfer throughout the scaffold, and hence supports the bone regeneration [3,4,5]. Moreover, the continuous surface and mean curvature of zero not only resemble the nature of the native trabecular bone [9] but also alleviate stress concentration comparing to scaffolds constructed with straight struts connected to sharp joints [10]. In specific, the network-phase gyroid is recognized as a practical geometrical design of BTE scaffold, owing to its interconnecting and controllable porosity that allows the union of the regenerated bone [7]. 

The materials of the scaffold are required for biocompatibility that induces no adverse effect to the biological system, biodegradability that allows the scaffold to be replaced by the regenerated bone, mechanical properties that allow load-bearing of scaffold and ideally, chemical/biological cues that stimulate the adhesion, proliferation and differentiation of osteoblasts [3,11,12]. In this regard, the phosphate glass/polylactic acid (PG/PLA) composites are considered promising for BTE scaffold. The phosphate glass consists of a hydrolysable glass network formed by the covalently cross-linked orthophosphate backbone that is ionically bonded with metallic cations. Upon incorporated into the biodegradable PLA matrix, the PG with high Young’s modulus (40–90 GPa [13,14]) enhances the mechanical properties of the composites. The hydrolytic degradation of PG releases Ca, Mg, and P that facilitate the deposition of hydroxyapatite on the surface of scaffolds to induce the osteoconductivity [15]. The presence of PG was reported to enhance differentiation of MG-63 osteoblast-like cells, while a PG/gelatin scaffold was found with comprehensive improvements of viability and proliferation profile of MG-63 cells and in vitro bioactivity over the gelatin-only counterparts, owing to the release of Ca, Mg, and P from the PG [16,17,18]. Recently, the PG/PLA composites were successfully fabricated via fused deposition modelling (FDM). Patient-specific fixation plates and porous BTE scaffold with the well-defined porous structure were successfully prepared [19,20]. Comparing to phosphate glass particles, the milled phosphate glass fibers (PGF) induced more effective mechanical reinforcement and a prolonged term of degradation of the corresponding PG/PLA composite [19].

Though the potential merits of PG/PLA composites towards bone tissue engineering are known, the degradation of PG produces PO_4_^3−^ that may compound with water to form phosphoric acid, resulting in acidification of degradation environment [21,22]. An acidic pH < 6.5 is reported to cause dramatically reduced activity and higher apoptosis rate of MC3T3-E1 murine pre-osteoblast [23,24]. Moreover, acidic pH may inhibit bone mineralization and enhance osteoclastic activities [25]. To prevent these effects from overwhelming the benefits of PG/PLA composite scaffolds, there is an imminent need of buffering the acidity due to phosphate glass degradation to avoid the negative impact thereof.

A possible solution is to incorporate a neutralizing agent to cope with the acidity generated by phosphate glass degradation [26]. Since the final goal is a long-term biodegradable biomedical implant, the neutralizing agent must be biocompatible and biodegradable in the human body, with controlled solubility in water to prolong the buffering period. Kobayashi et al. reported that adding 20 wt.% CaCO_3_ into the PGF/PLA composites buffered the acidity due to PGF degradation, but the degradation media (0.9% NaCl solution) was still considerably acidified (pH ~ 5) after 24 h of immersing the composite [27]. Hydroxyapatite (HA), a calcium and phosphate-containing mineral that has a similar chemical composition to human bone mineral, is also considered with the capacity of neutralizing the acidity due to the degradation of poly-α-hydroxy acids [28]. Conversely, it was reported that an increased HA loading in polylactic-co-glycolic acid (PLGA) led to even more pronounced pH reduction, due to the increased surface area of composites and a limited buffering ability of hydroxyapatite above the pH of 4.2 [26,29]. Previous work reported that the addition of HA into the PGF/PLA composite did not show any buffering effect, but led to a dramatically accelerated mechanical deterioration of composites instead [30].

It is considered that alkaline chemicals may be more effective to immediately neutralize the acidity. In recent years, magnesium wires and microparticles were investigated as neutralizing agents in PLA-based composites [31,32]. The mechanism is that magnesium reacts with water to produce hydrogen and magnesium hydroxide (Mg + H_2_O → H_2_ + Mg(OH)_2_), and the alkaline Mg(OH)_2_ may neutralize the acidity due to PLA degradation. However, the formation of hydrogen remains a safety concern as it may lead to complications such as subcutaneous emphysema and osteolytic lesions [33,34]. The use of magnesium oxide (MgO) was considered a safer choice, as it may be readily hydrated to produce magnesium hydroxide (MgO + H_2_O → Mg(OH)_2_) without generating hydrogen [35]. Recent work reported that by adding 3 wt.% of MgO nanoparticles, the additive-manufactured MgO/PLA scaffolds induced increased pH of degradation media and more importantly, an enhanced proliferation profile of MC3T3-E1 murine osteoblasts, possibly owing to the basicity at the scaffold surface that favors the growth of osteoblasts [23,36]. Regarding the addition of MgO into PG/PLA composites, the expected results include maintaining a neutral pH of the degradation media, meanwhile allowing Ca, P, and Mg to be released, thus minimizing the undesired cell death due to increased acidity and stimulate the bone-forming bioactivities of osteoblasts via the released elements.

In this study, PLA-based BTE scaffolds containing MgO, PGF or both were investigated. The scaffolds with network-phase gyroid design were additive-manufactured via FDM. Before degradation, the scaffolds were characterized for initial compressive properties. Next, the surface morphology, internal microscopic structure, mass changes of the scaffolds and the pH value, the concentration of Ca^2+^ and Mg^2+^ in degradation media were characterized after in vitro degradation. The results were then analyzed to understand the possible activities involved during the degradation of scaffolds, and the potential mechanism of MgO as a neutralizing agent in the (MgO + PGF)/PLA composite scaffolds.

## 2. Materials and Methods 

### 2.1. Materials

Phosphate glass fiber yarns with the chemical composition of P48-B12-Ca14-Mg17-Na1-Fe8 (the numbers indicate the molar percentage of each oxide of elements in the phosphate glass network) was supplied by Sinoma Co., Ltd, Nanjing, China. The fibers have a diameter of 9–13 μm. Rod-like magnesium oxide (MgO) microparticles were supplied by Kaishefeng^®^, Shanghai, China. The microscopic image of MgO was shown in Figure S1. The polylactic acid of Ingeo^®^ 4032D (Natureworks^®^, Minnetonka, MN, United States) was used in granule form. Tris(hydroxymethyl)aminomethane and 1.0 mol/L hydrochloric acid (HCl) aqueous solution were supplied by Sinopharm Chemical Reagents Co., Ltd, Shanghai, China. 1.0 mol/L nitric acid (HNO_3_) solution and lanthanum chloride (LaCl_3_) were supplied by Aladdin Reagents, Shanghai, China. 1000 μg/mL standard solutions of Ca^2+^ and Mg^2+^ were supplied by Guobiao (Beijing) Testing and Certificate Co., Ltd, Beijing, China.

### 2.2. Preparation of Feedstocks for FDM Additive Manufacturing

The FDM feedstocks for FDM were prepared by melt compounding–melt extrusion. Before melt compounding, the polylactic acid pellets, MgO and short-chopped PGF (~5 mm) were dried in an oven at 80 °C (polylactic acid) or 100 °C (MgO and PGF) for 3 h. The polylactic acid, MgO and PGF were then weighed according to specification in Table 1 and added into the mixing chamber of an internal mixer (ZG-5L, Zhenggong^®^, Dongguan, China). The materials were preheated under 205 °C for 5 min, followed by melt mixing under 205 °C, 20 rpm blades rotation for 10 min to produce free-form composite globs. The free-form composites were then pulverized into irregularly-shaped granules using a plastic pulverizer (DJT300, Dingjun^®^, Ningbo, China) equipped with a Ø 5 mm sieve.

The filament feedstocks for FDM were prepared by single screw extrusion. After drying in an oven for 80 °C for 2 h, the irregularly-shaped granules were added into the single-screw filament extruder (Model C-2, Wellzoom^®^, Shenzhen, China) equipped with a Ø 1.8 mm extrusion die. The temperatures of the preheat zone and the extrusion die were 200 °C and 190 °C, respectively, and the screw rotation rate was 30 rpm to produce filaments with a diameter of Ø 1.75 ± 0.20 mm. 

### 2.3. Design of Gyroid Porous Structure

**Figure 1:**
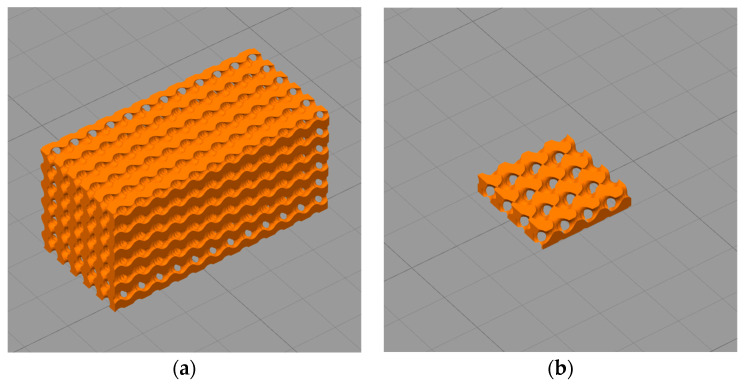
(**a**) Gyroid scaffolds intended for the compressive test, with the 12 mm × 25 mm plane of scaffolds was placed flat on the build plate; (**b**) Gyroid scaffold intended for in vitro degradation. The models were displayed in the Simplify3D slicing software, with the width of square grids equals to 1 cm.

The gyroid models with a size of 12 mm × 12 mm × 25 mm and 10 mm × 10 mm × 2 mm were designed using MathMod (Version 9.1, ParisoLab Inc., Montreal, Canada). The diameter of the pore channel in both gyroid models was 0.50 mm and the overall porosity was 50%. To obtain network-phase gyroid parts, the gyroid models were further processed using FreeCAD (Version 0.18, freeware). A rectangular, solid box with the same length, width, height and centroid as the gyroid model was generated. As the gyroid separated the box into two regions, the Boolean fragments of the box and gyroid were selectively removed so that only one of the regions was maintained to acquire the network-phase gyroid part. The 12 mm × 12 mm × 25 mm part was intended for compressive testing, while the 10 mm × 10 mm × 2 mm network-phase gyroid part was designated for in-vitro degradation (see Figure 1).

### 2.4. Additive Manufacturing via Fused Deposition Modelling (FDM)

**Figure 2:**
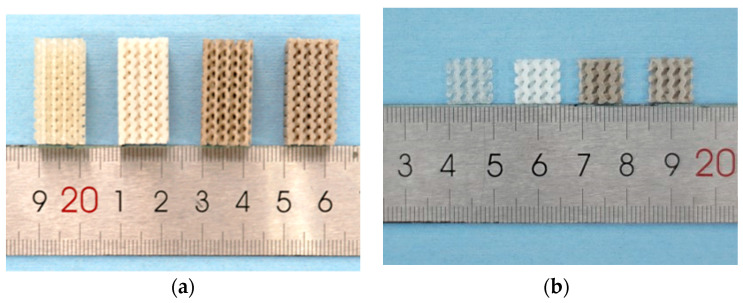
(**a**) Gyroid scaffolds intended for the compressive test; (**b**) Gyroid scaffolds intended for in-vitro degradation study. The material compositions of the four scaffolds (from left to right) are PLA, MgO/PLA, PGF/PLA, and (MgO + PGF)/PLA, respectively.

The Ø 1.75 ± 0.20 mm filaments obtained in Section 2.2 were fed into an FDM-based 3D printer (Ultimaker 2+, Ultimaker^®^, Utrecht, The Netherlands) to fabricate two types of porous parts specified in 2.3. For the scaffold used for in-vitro degradation, the 10 mm × 10 mm plane was horizontally laid on the build plate, whereas the compressive test specimen was laid on the build plate with its 12 mm × 25 mm plane (exactly as shown in Figure 1). Key parameters of the FDM process were listed in Table 2, and the produced scaffolds were shown in Figure 2.

### 2.5. Compressive Properties of the Scaffold

The compressive properties of specimens were characterized according to the ASTM D695-15 test method [37], using a universal testing machine (E45.105, MTS^®^, Eden Prairie, MN, United States) equipped with a 50 kN load cell. Compression at the rate of 1.3 mm/min was applied and the tests were carried out with 5 replicates for each material. The slope of the linear section of the stress–strain curve was determined as compressive modulus, whereas the peak compressive stress was determined as the compressive strength. The compressive tests were terminated automatically when the compressive load reduced to 50% of the peak compressive load recorded in the current test.

### 2.6. Fiber Length Measurement

The lengths of fibers contained in the PGF/PLA or (MgO + PGF)/PLA composites were measured using an optical microscope. A piece of 10 mm × 10 mm × 2 mm scaffold was added into 10 mL of dichloromethane to dissolve the PLA. The resultant viscous slurries containing PGFs were gently spread on the transparent glass slides. After drying in a fume hood for 30 min to evaporate the solvent, the transparent thin films containing PGFs were obtained on the glass slides. The films were then imaged under the optical microscope (NE930, Nexcope^®^, Ningbo, China). For each material, more than 1500 fibers were measured for their lengths using the NexImage software (version 10.4.3, Nexcope^®^, Ningbo, China).

### 2.7. Scanning Electron Microscopy (SEM) and Energy-Dispersive X-ray Spectroscopy (EDS)

The top surfaces, freeze-fractured surfaces obtained by bending the scaffolds immersed in liquid nitrogen, and the sediments produced after degradation of PGF/PLA scaffolds were imaged via SEM and analyzed for their elemental composition using EDS. The samples were first sputter-coated with 6 nm gold layer using the vacuum coating system (SCD-500, Leica^®^, Wetzlar, Germany), and then imaged using the scanning electron microscope (Sigma VP, Zeiss^®^, Oberkochen, Germany). EDS spectra were acquired using the X-act silicon drift detector (Oxford Instruments^®^, Abington-on-Thames, United Kingdom), under the accelerating voltage of 15 kV.

### 2.8. In-Vitro Degradation in Tris-HCl Buffer

The in-vitro degradation of scaffolds was performed in 0.05 mol/L Tris-HCl buffer with the pH adjusted to 7.40 ± 0.05 via the addition of 1.0 mol/L HCl solution. The 10 mm × 10 mm × 2 mm scaffolds were weighed for initial mass (M_i_) and added into the polypropylene centrifuge tubes filled with 10 mL of Tris-HCl. The centrifuge tubes were stored under 37 °C for up to 14 days, during which the immersion media was never refreshed. On Day 1 3, 7, 10, and 14, the scaffolds were taken out, blot dried with tissue paper and weighed for their moisture-containing mass (M_w_). After weighing, the scaffolds were dried in an oven for 24 h under 65 °C to remove the moisture. The mass after drying (M_d_) were then measured. The water uptake of scaffolds and the mass change of scaffolds after drying were calculated based on the equations reported elsewhere [38]:

Water Uptake = [(M_w_ − M_d_)/M_d_] × 100% (1)

Mass change = [(M_d_ − M_i_)/M_d_] × 100% (2)

In addition, the pH values of Tris-HCl immersing scaffolds at the abovementioned time points were characterized using the FE28 pH meter equipped with LE-460 electrode (Mettler-Toledo^®^, Greifensee, Swiss). Five replicates of scaffolds and immersion media were tested for each type of scaffold material.

### 2.9. Concentration of Mg^2+^ and Ca^2+^ in Degradation Media

The concentrations of Mg^2+^ and Ca^2+^ in degradation media were characterized via flame atomic absorption spectroscopy (AAS). Before the test, the degradation media was extracted and filtered with a syringe equipped with a 0.45 μm syringe filter. The filtered degradation media was then diluted to produce specimen solutions containing degradation media, 0.2 mol/L HNO_3_ and 0.01 mol/L LaCl_3_. Standard curve solutions of Mg^2+^ and Ca^2+^ were also prepared with the same concentration of HNO_3_ and LaCl_3_. The flame AAS tests of the specimen solutions were then carried out in air/acetylene flame using the atomic absorption spectrometer (iCE3500, Thermo Fischer Scientific^®^, Cambridge, United Kingdom).

### 2.10. Statistical Analysis

Unpaired *t*-test of compressive properties was carried out using the GraphPad Prism software (Version 8.2.1, GraphPad Software^®^, San Diego, CA, United States).

## 3. Results

### 3.1. Compressive Properties of Scaffolds

**Figure 3:**
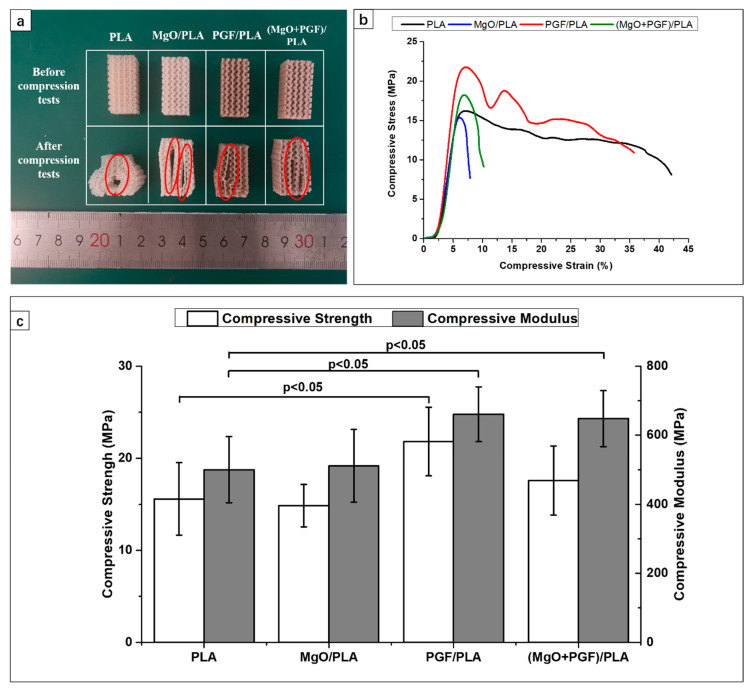
(**a**) Photograph of gyroid scaffolds before and after compression tests. Cracks propagated along the interlayer boundaries were highlighted with ovals; (**b**) Typical stress–strain curves of the gyroid scaffolds obtained from the compressive tests; (**c**) Summary of compressive strength and compressive modulus of the scaffold, with error bars representing the standard deviation. Five replicates were tested for each material. “*p* < 0.05” indicates statistical significance as analyzed based on unpaired *t*-tests.

Figure 3a displays the stress–strain curves of scaffolds under compressive loads. All specimens underwent elastic deformation at the beginning, then became yielded to reach maximum compressive stress, followed by a gradual reduction of stress against increasing deformation. The fluctuation of compressive stress of PGF/PLA composite was due to rupture and densification of porous gyroid structure. The breaking of PLA and PGF/PLA specimens were detected at a strain of over 35%. In contrast, the compressive stress of MgO-containing scaffolds reduced rapidly after the peak stress, and the break was soon detected at strains within 10%. Photograph of scaffolds after compressive testing is shown as Figure 3b. Cracks propagated along the boundary of adjacent layers can be seen in the central region of scaffolds. Deposited structures next to the crack were considerably deformed and ruptured.

The compressive strength and modulus of different scaffolds are summarized in Figure 3c. The average compressive strength and modulus of PLA scaffolds were 15.59 ± 3.59 MPa and 500.59 ± 91.31 MPa, respectively. The addition of 2 wt.% of MgO into the PLA matrix resulted in marginally lower compressive strength (14.86 ± 2.30 MPa) and enhanced compressive modulus (511.14 ± 105.04 MPa). The compressive strength (21.81 ± 3.72 MPa) and modulus (660.77 ± 79.15 MPa) were substantially higher for the PGF/PLA composite scaffolds. For the ternary (MgO + PGF)/PLA composite scaffold, the compressive strength (17.59 ± 3.75 MPa) deteriorated and reached an intermediate level between the two binary composite scaffolds, whereas the compressive modulus (648.14 ± 81.12 MPa) was close to that of PGF/PLA.

### 3.2. Fiber Length Measurements

**Figure 4:**
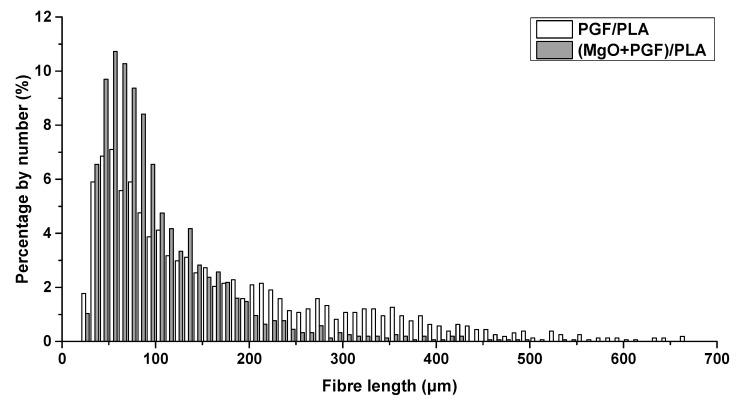
Distribution of the length of PGFs in PGF/PLA and (MgO + PGF)/PLA composite scaf.

Figure 4 shows the distributions of fiber length in PGF/PLA and (MgO + PGF)/PLA composite scaffolds. Statistics showed that the PGFs within PGF/PLA composite had an average length of 167.28 ± 131.71 μm and a median of 123.11 μm, in contrast to the average length of 103.54 ± 70.68 μm and median of 82.23 μm as measured from the PGF within the (MgO + PGF)/PLA composite. In summary, the PG fibers in the PGF/PLA composite were longer than those in (MgO + PGF)/PLA composite. 

### 3.3. Change of Size, Internal Structure, and Surface Morphologies of Scaffolds

**Figure 5:**
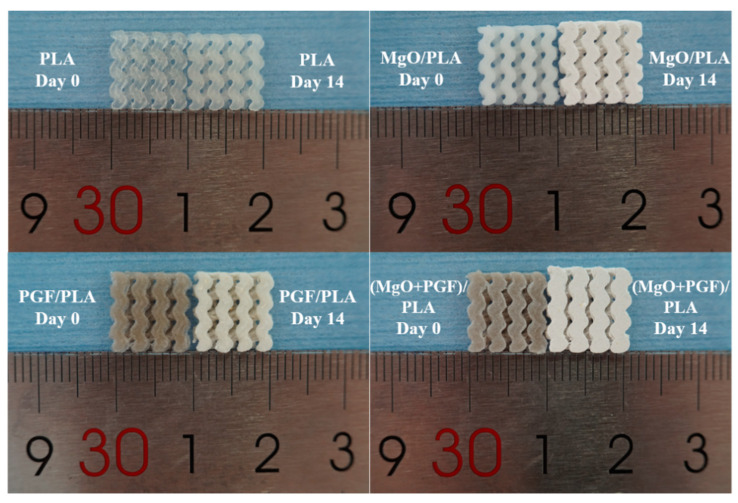
Photograph of scaffolds after 14 days of degradation.

Figure 5 displays the scaffolds after 14 days of degradation. The composite scaffolds containing MgO underwent size expansion, whereas the sizes of PLA and PGF/PLA scaffolds remained relatively stable after immersion.

**Figure 6:**
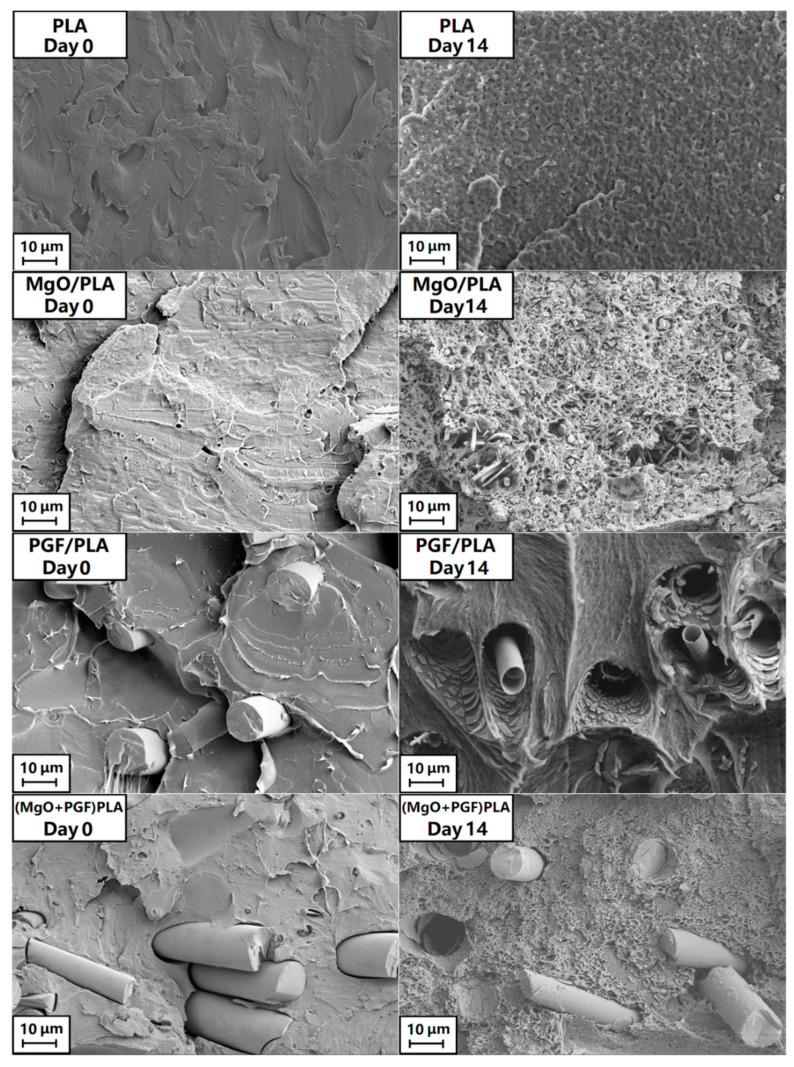
The freeze-fractured surfaces of different scaffolds before and after 14 days of in vitro degradation in Tris-HCl under 37 °C.

Figure 6 shows the fracture surfaces of four different specimen types before and after 14 days of in-vitro degradation. The Day 14 PLA scaffold specimen exhibited shallow pits which were evenly distributed across the fractured surface. The MgO/PLA composite at the same time point was less regular, with significantly higher porosity and dis-bonded MgO fragments visible as rods and particulate (see also Figure S2). The PGF/PLA composite after 14 days exhibited cylindrical PGFs sheaths and fragments thereof, suggesting an intensive coring or tunneling mode of PGF degradation. It is interesting to note that when MgO was added to the PGF/PLA composite the coring phenomenon was eliminated and the PG fibers remained in apparently good condition and were embedded in the matrix, which showed micron-level pitting across the fractured surface (see also Figure S3).

**Figure 7:**
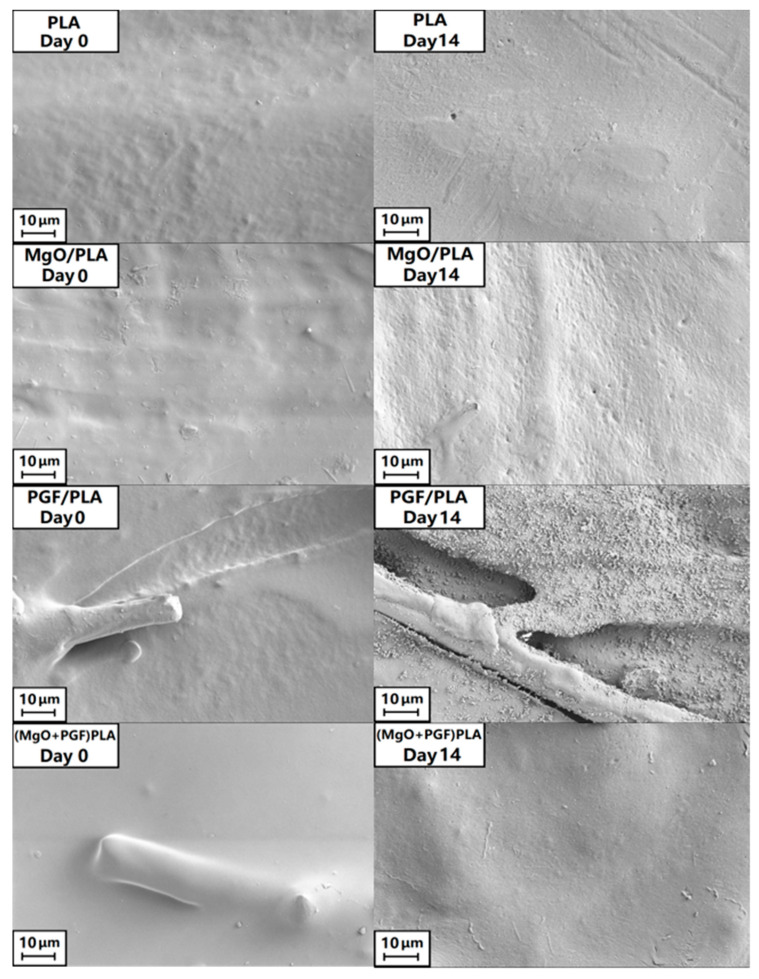
SEM image of the top surface of different scaffolds before and after 14 days of in vitro degradation in Tris-HCl under 37 °C.

**Figure 8:**
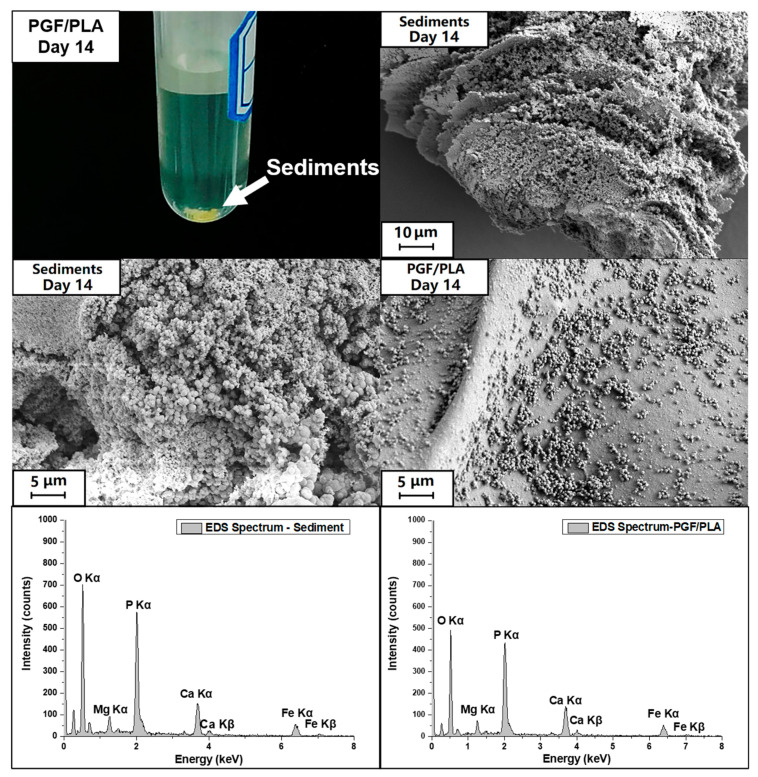
Photograph showing the yellow sediments in the degradation media immersed with PGF/PLA for 14 days, and the SEM images as well as the Energy-dispersive X-ray Spectroscopy (EDS) spectra of both the sediments and the top surfaces of PGF/PLA composite scaffolds.

The top surfaces of the scaffolds are shown in Figure 7. The surfaces of all non-degraded scaffolds were generally smooth, with both specimens containing PGF showing that the fibers were encapsulated by PLA. After 14 days of degradation, the surfaces for PLA and (MgO + PGF)/PLA composite did not show significant difference. Small pits were seen on the surface of MgO/PLA composite. The top surface of the PGF/PLA scaffold, however, was extensively disrupted with evidence of precipitates. Further characterization under SEM/EDS revealed that the precipitates were globular particles containing phosphate, magnesium, calcium, and iron, see Figure 8 and Figure S4.

Yellow sediments were exclusively observed in the degradation media of PGF/PLA composite scaffolds, see Figure 8. The sediment showed a multi-laminar morphology, with the surface densely covered with globular particles that were similar to the globular precipitates on the surface of degraded PGF/PLA scaffold (see also Figure S4). The EDS analysis showed that the sediments also contained calcium, magnesium, iron, and phosphate, all of which were released from the PGF.

### 3.4. Water Uptake and Mass Change of Scaffolds

**Figure 9:**
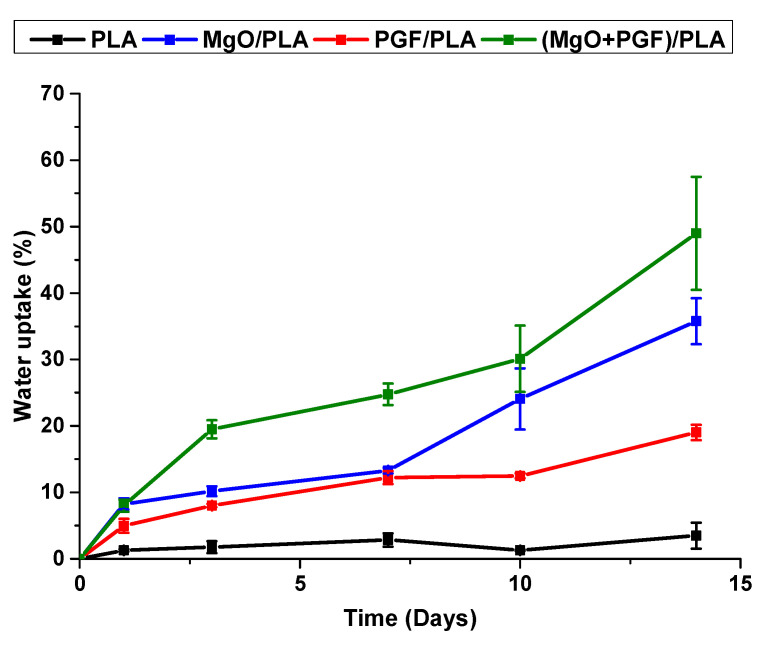
Water uptake profile of scaffolds during 14 days of in vitro degradation in Tris-HCl buffer under 37 °C.

Figure 9 presents the water uptake profile of the scaffolds. The water uptake of PLA was insignificant and fluctuated between 1% and 3% during the degradation study. More pronounced water uptake was seen in the binary composites. The MgO/PLA and PGF/PLA composite scaffolds remained similar to each other by Day 7, upon which the MgO/PLA scaffolds showed more pronounced water uptake. From Day 3 on, the water uptake percentages of the (MgO + PGF)/PLA composite scaffolds remained the highest among the tested materials and increased dramatically between Day 10 and Day 14.

**Figure 10:**
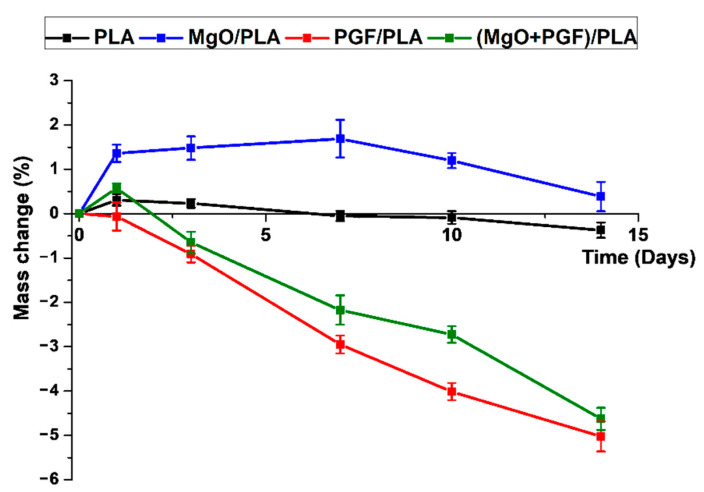
Mass change profile of scaffolds during 14 days of in vitro degradation in Tris-HCl buffer under 37 °C.

The mass change profile of scaffolds after drying is shown in Figure 10. The mass of PLA scaffolds increased by 0.31% on Day 1, followed by gradual reduction to −0.37% on Day 14, indicating a marginal mass loss after degradation. The MgO/PLA scaffolds showed increased mass in this study. A turning point was Day 7, upon which the extent of increased mass started to reduce, indicating that the activities causing mass loss outperformed the activities that lead to mass increment. The PGF/PLA composite showed a progressive mass loss during the study. For the (MgO + PGF)/PLA composite scaffolds, the mass increased slightly on Day 1, after which the mass loss became increasingly apparent

### 3.5. pH of Degradation Media (Tris-HCl Buffer)

**Figure 11:**
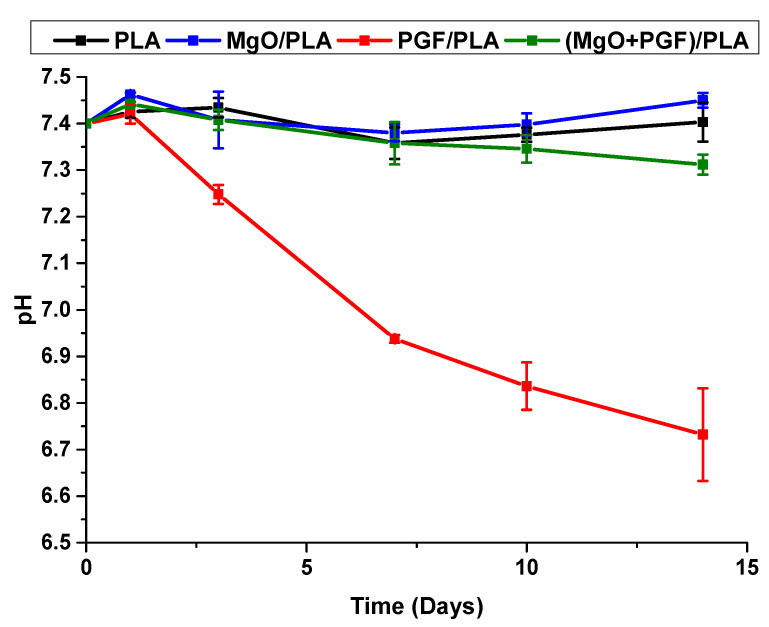
pH profile of the Tris-HCl immersed with different scaffolds during 14 days of in vitro degradation.

The average pH values of degradation media (Tris-HCl) immersing different scaffolds are shown in Figure 11. For PLA and MgO/PLA scaffolds, the pH maintained around 7.4 during the whole degradation period with no obvious variation. Continuous pH reduction since Day 3 was observed from the PGF/PLA composite and the pH dropped to 6.73 on Day 14. In contrast, the pH of ternary (MgO + PGF)/PLA composite dropped marginally to 7.31 on Day 14, remained neutral and close to the physiologic pH of 7.4.

### 3.6. Ion Release after Degradation

**Figure 12:**
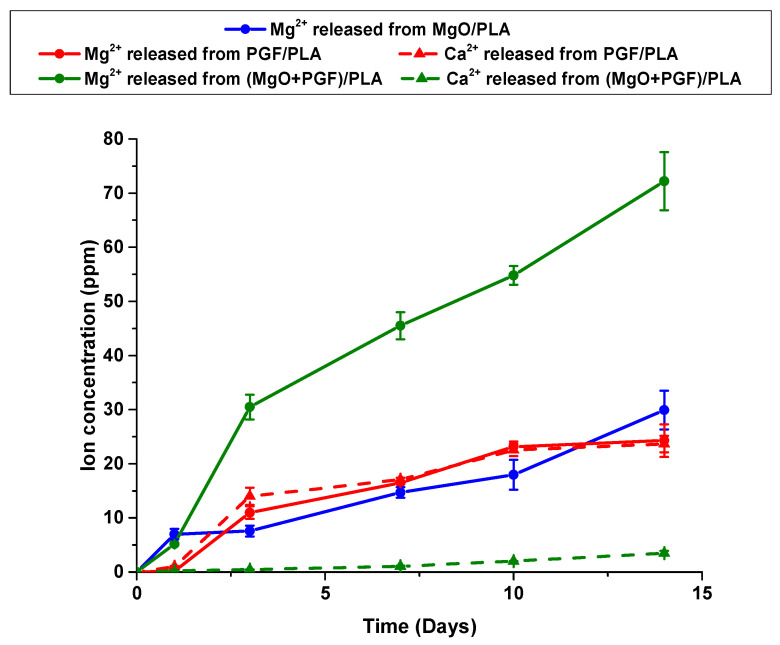
Ion concentration profile in the degradation media immersed with different scaffolds.

Ion concentrations in the degradation media are shown in Figure 12. Rapid release of Mg^2+^ from MgO/PLA composite was seen on Day 1, after which the Mg^2+^ concentration increased steadily. Almost no Mg^2+^ was leached from PGF/PLA composite within the first day, however, the Mg^2+^ concentration quickly increased to 10 ppm on Day 3 and increased steadily until Day 10, followed by attenuated growth by Day 14. Concerning the (MgO + PGF)/PLA composite, the Mg^2+^ concentration increased sharply within the first three days, followed by an almost-linear increment in the rest of the study, with the Mg^2+^ concentration remained higher than the sum of Mg^2+^ concentrations of the two binary composites. 

The dramatic difference in the amount of Ca^2+^ released from the PGF/PLA and (MgO + PGF)/PLA composites was also shown in Figure 12. For the PGF/PLA composite, the change of Ca^2+^ concentration was almost identical to that of the Mg^2+^ concentration. In contrast, the concentration of Ca^2+^ released from (MgO + PGF)/PLA composite increased steadily in the whole period but remained one order lower than that of the PGF/PLA composite.

## 4. Discussion

The performance of a scaffold to facilitate bone regeneration is greatly dependent on its structural and material properties. Regarding the structure, the network-phase gyroid with 0.50 mm pores investigated in this work is considered to provide a continuous surface for cell adhesion, adequate porosity, and appropriate size that favor mass transfer, bone ingrowth, and angiogenesis. Though the PG/PLA composites were regarded promising for bone tissue engineering application, the degradation media would be inevitably acidified due to degradation of both components, and this problem has not been properly addressed in previous studies. This work investigated the use of MgO to buffer the acidity after composite degradation. The scaffolds were comprehensively characterized to understand their potential in bone tissue engineering.

Regardless of material composition, all scaffolds underwent elastic deformation, yielding, and rupture during the compressive tests. The activities under compression were different from others’ works, where the gyroid scaffolds underwent elastic deformation (linear stress increment), buckling/densification (insignificant stress increment), and finally deformation after densification along with increased compressive strain (substantial and non-linear stress increment) [10,39]. Meanwhile, the cracks propagated along the boundary of FDM-deposited layers were observed. These findings collectively indicate a failure mode of debonding between the adjacent FDM-deposited layers. On the one hand, the compressive load was parallel with the interlayer bonding of scaffolds, and this might induce shearing force between adjacent layers. On the other hand, the strength of interlayer bonding of an FDM product is typically lower than that of the bulk materials, due to either poor interlayer contact or inadequate polymer diffusion [40]. Therefore, the interlayer debonding occurred before buckling of the scaffold.

With the outstanding tensile strength of 1180 MPa and tensile modulus of 63 GPa, the addition of the milled PGF would reasonably enhance the mechanical properties of resultant PGF/PLA composite [14]. The addition of MgO did not significantly alter the compressive modulus and strength of composites, whereas the strain-at-break reduced apparently. These findings indicate poor bonding between the MgO and PLA [41].

The compressive modulus of (MgO + PGF)/PLA composite was lower than the binary PGF/PLA scaffolds. This could be attributed to the lower fiber length that typically leads to inferior Young’s modulus of short-fiber-reinforced composites. The difference of fiber length within the two composites was possibly due to different viscosity of polymer melts during the melt-compounding. The length of fibers in a viscous liquid subjected to laminar shear is negatively correlated to the product of shear rate and viscosity of liquid [42,43]. It was likely that the addition of MgO resulted in a higher viscosity of the polymer melts than the non-filled PLA, leading to more pronounced bending, buckling, and thus length reduction of fibers. Consequently, the resultant composite possessed the inferior compressive modulus.

The bone tissue engineering scaffolds are typically required to possess mechanical properties that are comparable with trabecular bones, which have the typical compressive strength and compressive modulus of 0.1–30 MPa and 10–3000 MPa, respectively [44,45]. The compressive mechanical properties of the gyroid scaffolds studied here are comparable with those of the human trabecular bones. 

**Figure 13:**
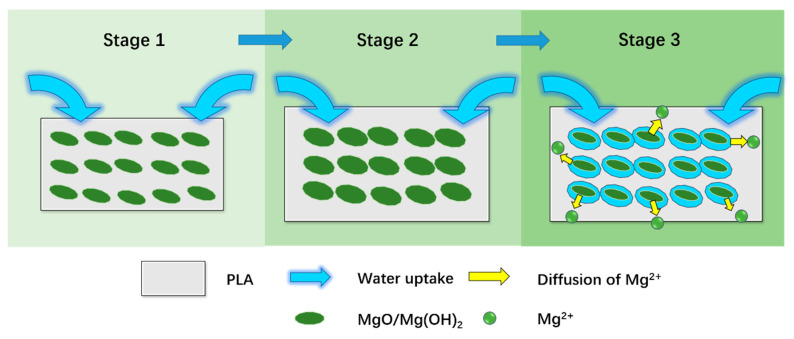
Schematic representation of the degradation of MgO/PLA composite scaffolds.

The discernible changes in the scaffold size provoked us to look into the internal structure of the degraded composite scaffolds. The scaffolds containing MgO expanded in their sizes due to the hydration of MgO, which absorbs water to produce Mg(OH)_2_ and results in a theoretical volumetric expansion by more than 200% [46]. As illustrated in Figure 13, at the beginning of degradation (Stage 1), water was first absorbed into the composites, leading to the expansion of MgO. The expansion of MgO then resulted in a macro expansion of the PLA scaffolds (Stage 2). Finally, at Stage 3, the dissolution of MgO and/or Mg(OH)_2_, as well as the erosion of PLA, resulted in porosity within the composites. The increased porosity allowed more water to be absorbed and thus explained the increased water uptake percentages after Day 10. The pores were retained in the composite after drying in the oven, corresponding to the voids and cavities across the freeze-fractured surface of the MgO/PLA scaffold. 

Changes of the surface morphology after degradation were rather insignificant, with the only exception being the PGF/PLA composite, upon which the globular precipitates containing P, Mg, Ca, and Fe were extensively found. These globular precipitates resembled those of the yellow sediment in both morphology and elemental composition. These findings were similarly reported by Shah Mohammadi et al, who claimed that the precipitates contained brushite (dicalcium phosphate dihydrate, CaHPO_4_·2H_2_O), a yellow calcium phosphate mineral favorably formed in an acidic environment [47,48]. Alani et al. reported that precipitation formed after the degradation of PG/PCL composites was only found on the composites containing fast-degrading PG, which produced a large number of ions after degradation, leading to supersaturation of Ca^2+^ and PO4^3−^ and resultant mineral precipitation [49,50]. The formation of precipitates indicated that the PGF within the PGF/PLA composite scaffolds underwent substantial degradation in a short period.

The water absorption and degradation of composite scaffolds both led to variation in the mass of the scaffolds. There are three mechanisms of water absorption in polymer-based composites: the intrusion of water molecules into the (1) free spaces between polymer chains; (2) gaps and defects at the reinforcement-matrix interfaces, and (3) the cavities created after the plastic deformation of composites or erosion/detachment of fillers [51,52]. A relatively low (1–3%) water uptake percentage in PLA scaffolds inferred that the water intrusion into free spaces between PLA chains was rather limited throughout the whole study. In contrast, the water uptake by ~8% in MgO/PLA composite after 1 day of immersion was mainly due to water ingress at the gaps and defects the MgO-PLA interfaces. As the expansion, dissolution of MgO and erosion of PLA occurred, cavities were generated inside the composite, which might correlate to the more pronounced water uptake after Day 10. The activities occurred during the immersion of PGF/PLA composite were similar, with the water uptake mainly attributed to interfacial uptake at first, followed by the dissolution of PGF that created cavities inside the composite and enhanced water absorption. The lower water uptake percentage in PGF/PLA than the MgO/PLA composite was possibly due to a lower area at the reinforcement-matrix interface. This could be attributed to the much lower specific surface area of milled PGF, which were apparently larger than MgO microparticles. The abovementioned activities also took place during the degradation of (MgO + PGF/PLA) composite. The higher water uptake of this composite was possibly related to the more pronounced dissolution of MgO that neutralized the acidity. This activity contributed to increased free spaces within the composite to take up water. Moreover, as the composite contained both MgO and PGF, the volume of gaps and defects at the reinforcement-matrix interface was considered to be the greatest, and the capacity for water absorption at the interface would be reasonably large.

The mass change of the dried composite scaffold revealed whether the mass of the scaffolds increased (showing positive mass change) or reduced (showing negative mass change) after degradation. The mass change profile of PLA suggests that little PLA eroded after 14 days of degradation. The MgO/PLA composite scaffolds showed an increasing mass from Day 1 to Day 7. Such a mass increment could be attributed to the absorption of water molecules on the MgO surface, as the water was tightly bound and cannot be removed below 200 °C [53]. The hydration of MgO would take up water to produce Mg(OH)_2_ and also led to the increased mass of composite [46,54]. From Day 7 on, the mass loss activities became more apparent than the abovementioned activities that lead to increased mass. This was possibly related to the dissolution of MgO and/or Mg(OH)_2_. Moreover, the produced Mg(OH)_2_ was able to induce an alkaline microenvironment, which could facilitate the PLA degradation (possibly corresponding to the pits at the surface [55] and cavities inside the composite [56]), and thus enhance the mass loss [57]. The mass of PGF/PLA composite kept reducing after Day 1, suggesting the progressive hydrolytic degradation of PGF. Regarding the (MgO + PGF)/PLA composite, the extensive pits at the fractured surface also indicated the degradation of PLA. While the dissolution of MgO and PGF also contributed to the reduced mass after Day 3, analysis over the Mg^2+^ and Ca^2+^ concentration is required to elaborate how much did each of the components dissolved.

The AAS results showed that Mg^2+^ was continuously released from the MgO/PLA composite due to dissolution of either MgO or its hydration product. This finding provides evidence for dissolution of MgO or its hydration product (Mg(OH)_2_). An attenuated increment of Mg^2+^ was observed after Day 10 of the degradation of the PGF/PLA composite. This was possibly correlated to the sediments and the globular precipitates on the surface of PGF/PLA scaffolds, which were produced under a supersaturated ionic concentration equilibrated with the PGF degradation and mineral precipitation. The degradation of (MgO + PGF)/PLA composite produced a substantially greater amount of Mg^2+^ without sings of equilibrium. Since both the MgO and PGF degradation will contribute to Mg^2+^ release, the change of Mg^2+^ may not distinguish the amount of MgO and PGF degraded from the ternary composite.

The change of Ca^2+^ concentration revealed the degradation of PGF from the composite scaffolds. For the PGF/PLA composite, the change of Ca^2+^ concentration resembled the trend of Mg^2+^ concentration. In contrast, the Ca^2+^ released from (MgO + PGF)/PLA was 90% lower than the Mg^2+^ concentration at the same time. Together, the cation concentration and the absence of globular precipitation indicated that the PGF degradation in the (MgO + PGF)/PLA composite was considerably fewer than that in the PGF/PLA composite. Therefore, the high Mg^2+^ concentration was due to the substantial dissolution of MgO/Mg(OH)_2_ in the (MgO + PGF)/PLA composite. These results also indicate that the dissolution of MgO/Mg(OH)_2_ considerably contributed to the mass loss of (MgO + PGF)/PLA.

It is known that the hydrolytic degradation of PGF and dissolution of MgO/Mg(OH)_2_ are both sensitive to the pH. The degradation rate of phosphate glass was lowest in neutral media but became greatly accelerated with increasing acidity of degradation environment [58,59]. Meanwhile, it was reported that the surface of MgO, which remained stable in the basic and neutral condition, was etched as the pH reduced to 6 [60], and the MgO was increasingly dissolved in an aqueous media with reduced pH [61]. In addition, the dissolution of Mg(OH)_2_ (Mg(OH)_2_ → Mg^2+^ + 2 OH^−^) is facilitated under high acidity, as the OH^−^ may be rapidly neutralized by the H^+^ in an acidic media.

**Figure 14:**
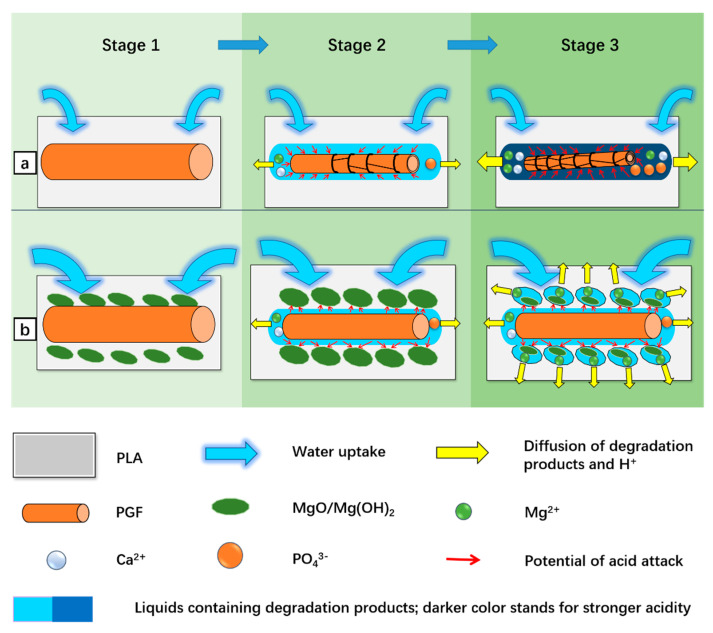
Schematic representation of the possible degradation mechanisms for different composites. (**a**) a mechanism for the binary PGF/PLA composite; (**b**) mechanism for the ternary (MgO + PGF/PLA) composite.

Based on the results, the possible activities involved in the degradation of PGF/PLA and (MgO + PGF)/PLA scaffolds are illustrated in Figure 14. At the beginning (Stage 1), water infused into the composite scaffolds, initiating the hydration and dissolution of MgO and PGF. Next (Stage 2), as the PG fibers were encapsulated inside the PLA, the degradation of PGF resulted in a local acidity at the PGF/PLA interface, which intensified the PGF degradation in return. The acidic compounds slowly diffused into degradation media and manifested in pH reduction. In contrast, the acidity due to PGF degradation was effectively neutralized by the surrounding MgO and/or Mg(OH)_2_ in the ternary composite, therefore preventing local acidity to aggravate the PGF degradation. Finally (Stage 3), as MgO and Mg(OH)_2_ were extensively consumed to neutralize the acidity, the Mg^2+^ was released into surrounding media and showed a reasonably higher concentration than that of the MgO/PLA composite.

In order to facilitate the bone repair, the BTE scaffolds must support the colonization of osteogenic cells but are also anticipated to provide biological and chemical cues to enhance the proliferation and osteoblastic differentiation of cells. The biodegradable scaffolds studied herein may promote bone repair activities by releasing calcium, magnesium, and not to forget, phosphorus to the degradation media. An increasing Ca^2+^ concentration within the range of 0.1–0.4 mmol/L (approximately 4–16 ppm) was reported to upregulate the proliferation activities and production of bone morphogenic protein−2 and −4 of human osteoblastic cells [62], while Maeno et al. found that a further increment of Ca^2+^ concentration below 6 mmol/L (240 ppm) would upregulate the osteocalcin production and calcium deposition of murine osteoblasts [63]. Meanwhile, human osteoblasts remained viable at a high Mg^2+^ concentration of 16 mmol/L (384 ppm), and an increasing level of Mg^2+^ concentration by 0.8 to 1.8 mmol/L (19.2–43.2 ppm) promoted osteogenesis of mesenchymal stem cells [64,65]. Moreover, the Ca and P released from PG are essential to the formation of bone-like apatite in the biological system and contribute to the osteoconductivity of scaffolds [66,67]. However, these beneficial effects would be compromised as an acidic pH not only adversely affects the activities of osteoblasts but also promotes the formation of brushite instead of the bone-like apatite [23,28]. Referring to the current study, the (MgO + PGF)/PLA composite was the only material that satisfied the requirements for maintaining a neutral pH after degradation, meanwhile releasing Mg, Ca, and P into the degradation media to potentially promote the bone-forming activities. Pre-clinical biological studies are needed to validate the applicability of scaffolds for BTE, including in vitro cell culture, and in vivo tests to evaluate biocompatibility, osteoconductivity and biodegradability in situ.

## 5. Conclusions

In this work, porous network-phase gyroid scaffolds were additive-manufactured via FDM, using four materials: PLA, MgO/PLA, PGF/PLA, and (MgO + PGF)/PLA. The contents of MgO and PGF fixed at 2 wt.% and 18 wt.%, respectively.

The compressive modulus and strength of scaffolds were considerably enhanced by PGF. The addition of MgO resulted in reduced strain-at-break of composites, indicating a weak MgO/PLA interface. In general, the compressive properties of the scaffolds were comparable with human trabecular bone.

SEM and EDS results revealed that the PGF within the PGF/PLA composite degraded significantly, evidenced by exfoliation of PGF and precipitation of globular particles containing supersaturated P, Ca, Mg, and Fe. These effects were not observed after the degradation of the (MgO + PGF)/PLA composite.

As expected, the addition of MgO neutralized the acidity due to PGF degradation. The degradation media of PGF/PLA composite scaffolds underwent apparent pH reduction from 7.40 to 6.73 after 14 days of conditioning, in contrast to the neutral pH (7.31) maintained after degradation of (MgO + PGF)/PLA. The possible mechanism of MgO as a neutralizing agent, as collectively inferred from SEM/EDS results, mass change of scaffolds, pH, and ion concentration profile of degradation media, was that the local acidity was buffered as the MgO reacted with acidic degradation products, therefore inhibiting the intensification of PGF degradation in an acidic environment.

In conclusion, the addition of MgO was able to neutralize the acidity due to PGF degradation and this has multiple benefits. First, the neutral pH would be more likely to favor tissue regrowth; secondly, the suppression of acidic degradation products avoids the potential auto-acceleration of glass degradation and would enable a longer period of retaining the structural integrity of the scaffold and releasing elements that favor bone growth. The network-phase gyroid (MgO + PGF)/PLA composite scaffold had the trabecular-bone-mimetic compressive properties and the ability to release Mg, Ca, and P along with degradation, showing good promise for bone tissue engineering application. Further studies would usefully investigate the responses of osteoblastic cells and bone tissue to these scaffolds, exploiting both their interesting geometric features as well as the facility to control local acidity and release therapeutic ions.

## Figures and Tables

**Table 1:** Sample codes and material compositions of filament feedstocks for additive manufacturing.

Sample Code	PLA (wt.%)	MgO (wt.%)	PGF (wt.%)
PLA	100	-	-
MgO/PLA	98	2	-
PGF/PLA	82	-	18
(MgO + PGF)/PLA	80	2	18

**Table 2:** Key parameters in the additive manufacturing process.

Parameters	Value/Setting
Nozzle diameter	0.4 mm
Layer height	0.15 mm
Build plate temperature	60 °C
Nozzle temperature	190 °C
Default printing speed	1800 mm/min

## Data Availability

The data presented in this study are available on request from the corresponding author.

## Supplementary Materials

The following are available online at https://www.mdpi.com/2073-4360/13/2/270/s1, Figure S1: SEM image of MgO rod-like microparticles, Figure S2: Freeze-fractured surface of a MgO/PLA composite scaffold after 14 days of immersion in Tris-HCl buffer. The arrow points at the rod-like MgO nanoparticles, Figure S3: Freeze-fractured surface of a (MgO+PGF)/PLA composite scaffold after 14 days of immersion in Tris-HCl buffer, Figure S4: Top surface of a PGF/PLA composite scaffold after 14 days of immersion in Tris-HCl buffer (under a higher magnification of 5000×).
