# Supplementary material for: Additive-Manufactured Gyroid Scaffolds of Magnesium Oxide, Phosphate Glass Fiber and Polylactic Acid Composite for Bone Tissue Engineering
Source: Polymers (Basel). 2021 Jan 15;13(2):270. doi: 10.3390/polym13020270 (PMC7830155; doi:10.3390/polym13020270)
Supplement: Supplementary file 1 [file polymers-13-00270-s001.pdf]

Additive-manufactured Gyroid Scaffolds of Magnesium Oxide, Phosphate Glass Fiber and Polylactic acid Composite for Bone Tissue Engineering

Lizhe He [1], Xiaoling Liu [1,2,3,*] and Chris Rudd [4]

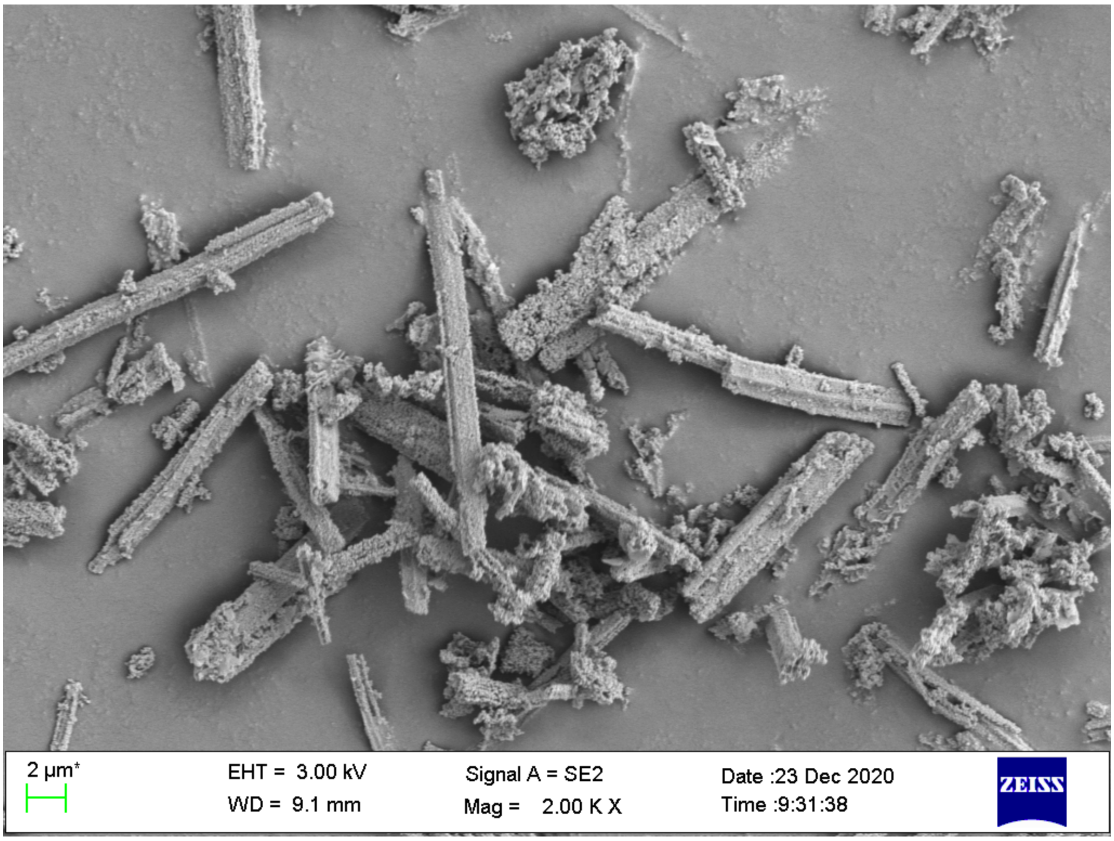

Figure **S1.** SEM image of MgO rod-like microparticles.

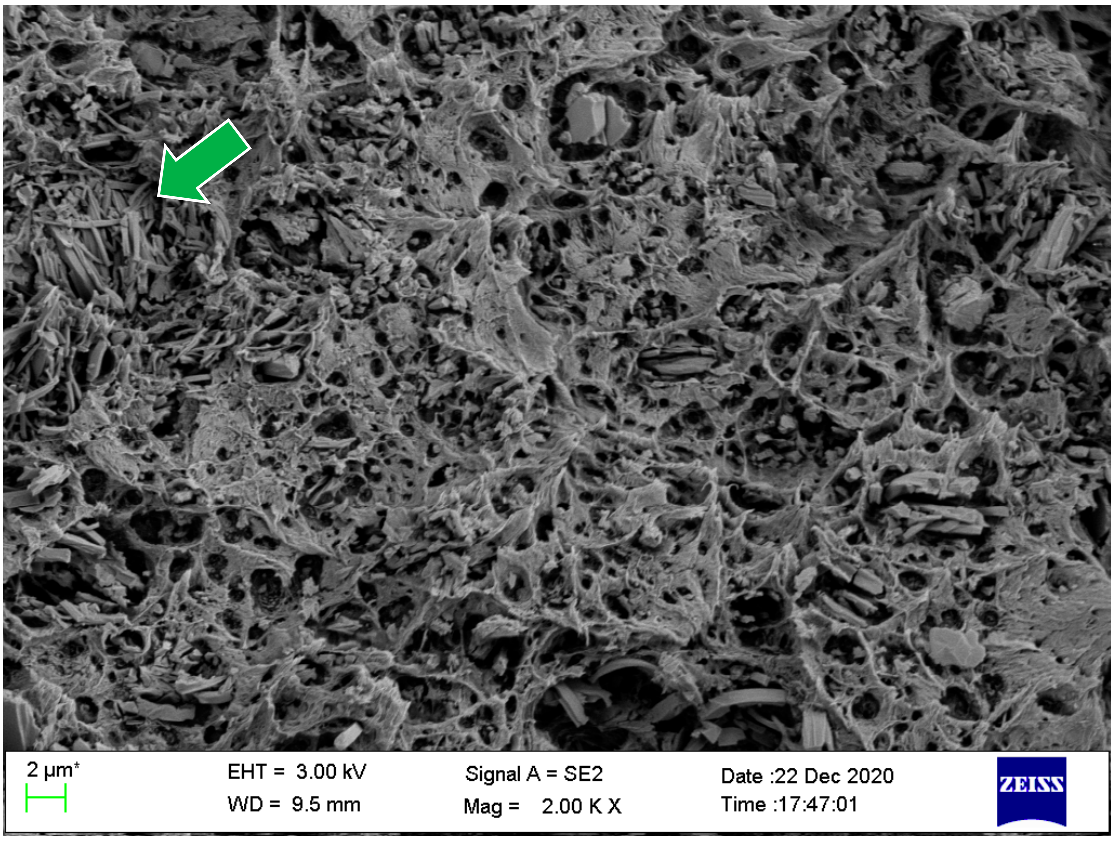

Figure **S2.** Freeze-fractured surface of a MgO/PLA composite scaffold after 14 days of immersion in Tris-HCl buffer. The arrow points at the rod-like MgO nanoparticles

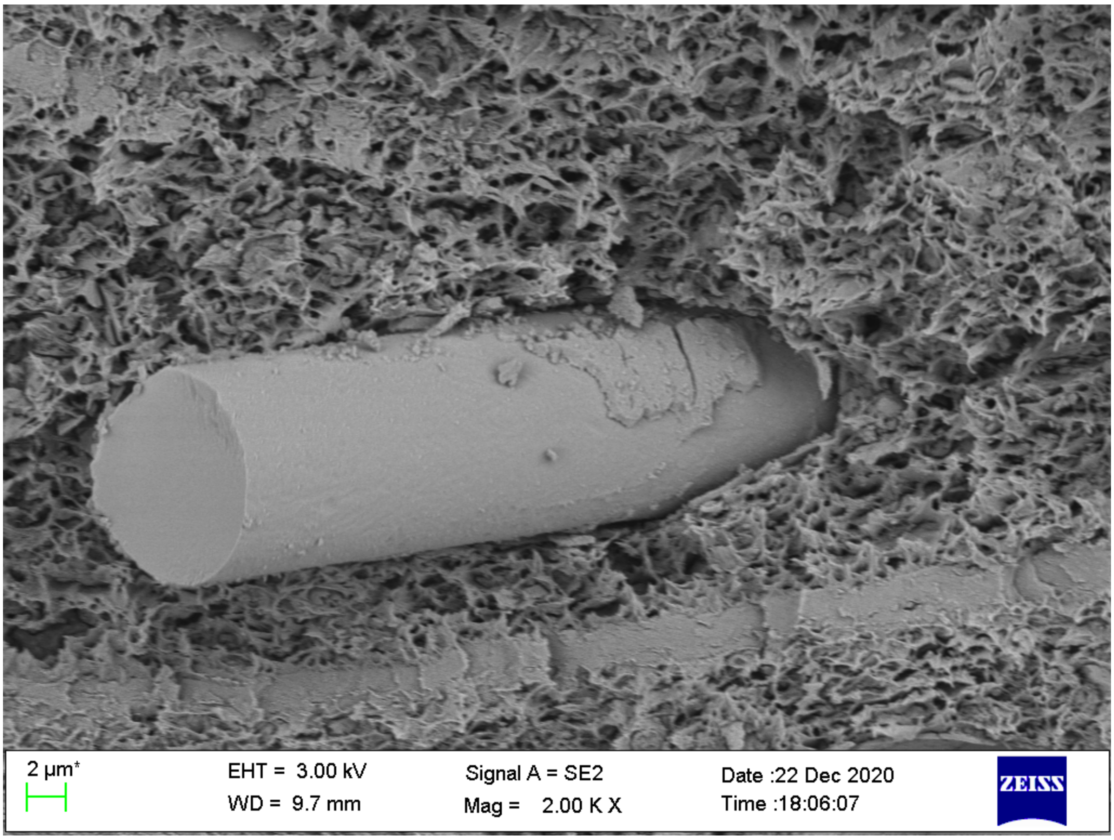

Figure **S3.** Freeze-fractured surface of a (MgO+PGF)/PLA composite scaffold after 14 days of immersion in Tris-HCl buffer.

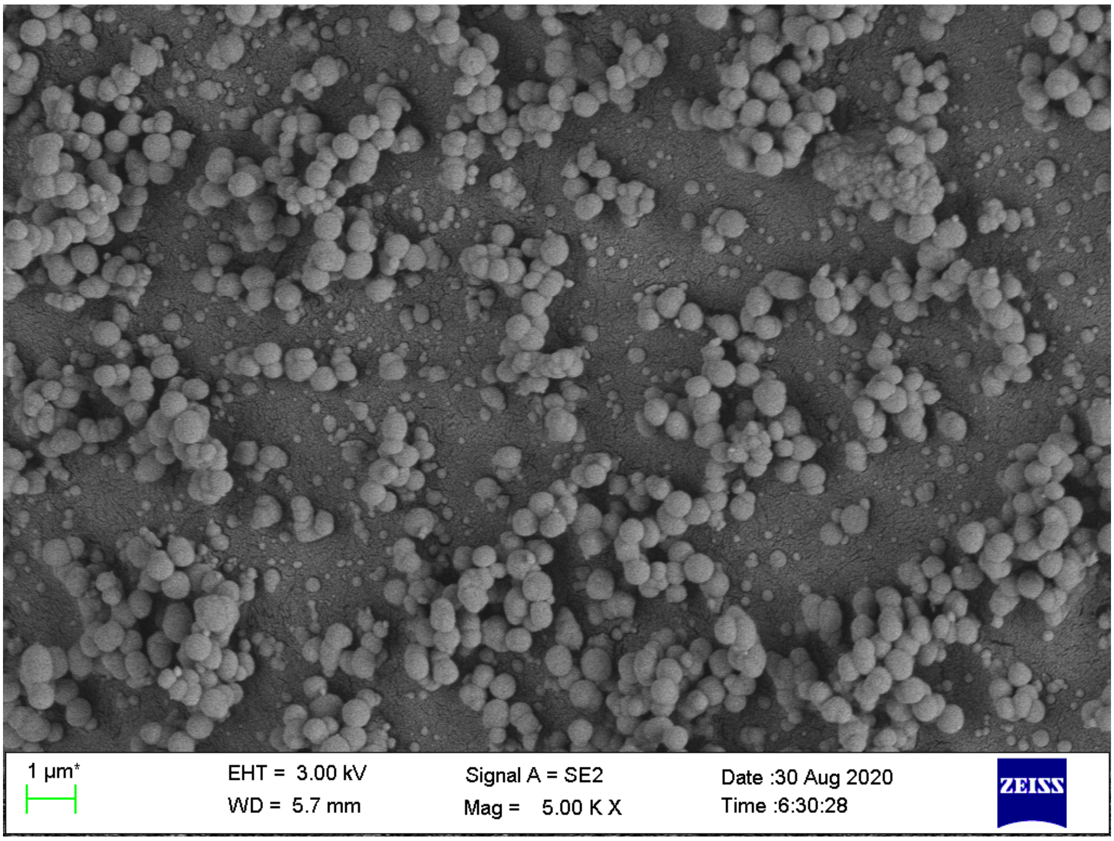

Figure **S4.** Top surface of a PGF/PLA composite scaffold after 14 days of immersion in Tris-HCl buffer (under a higher magnification of 5000x).
